# Indole intercepts the communication between enteropathogenic *E. coli* and *Vibrio cholerae*

**DOI:** 10.1080/19490976.2022.2138677

**Published:** 2022-11-03

**Authors:** Orna Gorelik, Alona Rogad, Lara Holoidovsky, Michael M. Meijler, Neta Sal-Man

**Affiliations:** a The Shraga Segal Department of Microbiology, Immunology, and Genetics, Faculty of Health Sciences, Ben-Gurion University of the Negev, Beer-Sheva, Israel; b Department of Chemistry, the National Institute for Biotechnology in the Negev Ben-Gurion University of the Negev, Be’er Sheva, Israel

**Keywords:** Bacterial virulence, bacterial communication, indole, autoinducer, CAI-1, EPEC, *Vibrio cholerae*

## Abstract

Reported numbers of diarrheal samples exhibiting co-infections or multiple infections, with two or more infectious agents, are rising, likely due to advances in bacterial diagnostic techniques. Bacterial species detected in these samples include *Vibrio cholerae* (*V. cholerae*) and enteropathogenic *Escherichia coli* (EPEC), which infect the small intestine and are associated with high mortality rates. It has previously been reported that EPEC exhibit enhanced virulence in the presence of *V. cholerae* owing to their ability to sense and respond to elevated concentrations of cholera autoinducer 1 (CAI-1), which is the primary quorum-sensing (QS) molecule produced by *V. cholerae*. In this study, we examined this interspecies bacterial communication in the presence of indole, a major microbiome-derived metabolite found at high concentrations in the human gut. Interestingly, we discovered that although indole did not affect bacterial growth or CAI-1 production, it impaired the ability of EPEC to enhance its virulence activity in response to the presence of *V. cholerae*. Furthermore, the co-culture of EPEC and *V. cholerae* in the presence of *B. thetaiotaomicron*, an indole-producing commensal bacteria, ablated the enhancement of EPEC virulence. Together, these results suggest that microbiome compositions or diets that influence indole gut concentrations may differentially impact the virulence of pathogens and their ability to sense and respond to competing bacteria.

## Introduction

According to the World Health Organization, diarrheal diseases remain one of the leading causes of death among children under the age of five in developing countries, causing more than 500,000 deaths annually. These infections have traditionally been attributed to a single infectious agent. However, improvements in diagnostic techniques have revealed that samples from diarrheal disease patients are often consistent with co-infection by two or more infectious agents.^1–3^ These multi-pathogen infections can appear in up to 60% of all tested samples, with *Escherichia coli, Vibrio cholerae* (*V. cholerae*), and *Shigella* species being the predominant pathogens observed in affected patients.^2–4^ These co- and multi-infections are commonly associated with more severe clinical symptoms, likely owing to the higher overall infectious load or the enhancement of the virulence of at least one of the infecting species.^4–6^

We have recently studied one such virulence enhancement mechanism by exploring the interplay between *V. cholerae* and enteropathogenic *E. coli* (EPEC), which are two of the primary infectious drivers of gastroenteritis. Specifically, we found that EPEC enhances its virulence in the presence of *V. cholerae* through its ability to detect elevated concentrations of cholera autoinducer 1 (CAI-1), which is the primary quorum-sensing (QS) molecule produced by *V. cholerae*.^7–9^ In *V. cholerae*, CAI-1 is synthesized by the CqsA enzyme, secreted to the extracellular environment, and its concentration rises as the size of the *V. cholerae* population increases. Once a threshold concentration has been reached, CAI-1 binds to the CqsS receptor on *V. cholerae* to alter the transcription of virulence factors and biofilm development-related genes.^8,9^

EPEC relies on the type III secretion system (T3SS) to infect host cells.^10^ The T3SS is a large protein transport complex that many other pathogenic gram-negative bacteria use to form a nano-syringe structure. The T3SS translocates effectors directly into host cells, where they interfere with crucial cellular processes that ultimately promote bacterial replication and transmission.^11–13^ In EPEC, the T3SS is encoded on a large 35-kbp chromosomal pathogenicity island, known as the locus of enterocyte effacement (LEE).^14^ The LEE consists of 41 genes, organized in seven operons (LEE1–LEE7), that encode structural proteins, regulators, and effector proteins.^13,15,16^ We discovered that EPEC T3SS activity, and hence its infection ability, are enhanced in response to CAI-1.^7^

EPEC and *V. cholera* co-infections occur in the small intestine,^17^ which is colonized by a diverse population of microorganisms collectively referred to as the microbiome.^18,19^ The gut microbiome regulates diverse physiological processes, such as food digestion and metabolite production, the maintenance of the gut mucosal barrier, and the prevention of pathogenic invasion.^18–22^ Microbiome-derived metabolites are essential for the regulation of the intestinal immune system and the maintenance of the gut microbiome homeostasis,^23–26^ thereby shaping human health and disease.^27–29^ In this study, we focused on indole, an amino-acid-derived metabolite produced from the degradation of tryptophan by a tryptophanase enzyme encoded by the tnaA gene mainly in commensal bacteria such as *Bacteroides thetaiotaomicron*.^23,25,26^ Indole concentrations are estimated to range as high as 1 mM in the human gastrointestinal tract.^25,30^ These high indole concentrations have been shown to decrease enterohemorrhagic *E. coli* (EHEC) motility, biofilm formation, adherence to epithelial cells, and virulence gene expression, in addition to enhancing drug resistance of *Salmonella enterica*.^25,31–33^

In this study, we characterized EPEC responses to the presence of *V. cholerae* under conditions that better simulate the small intestine and examined whether microbiome-derived indole can alter the communication between these two pathogens. Interestingly, we discovered that although indole did not affect the growth or CAI-1 production of *V. cholerae*, it neutralized the upregulation effect of *V. cholerae* on EPEC T3SS, either by acting as a strong virulence inhibitor or by interfering with the cross-talk between EPEC and *V. cholerae*. Overall, our results suggest that the microbiome can indirectly affect bacterial virulence by producing metabolites that attenuate pathogen virulence and intercept pathogen communication, thus suggesting a tight connection between commensal bacteria and pathogens virulence.

## Results

### Indole inhibits EPEC T3SS activity

**Figure 1.**
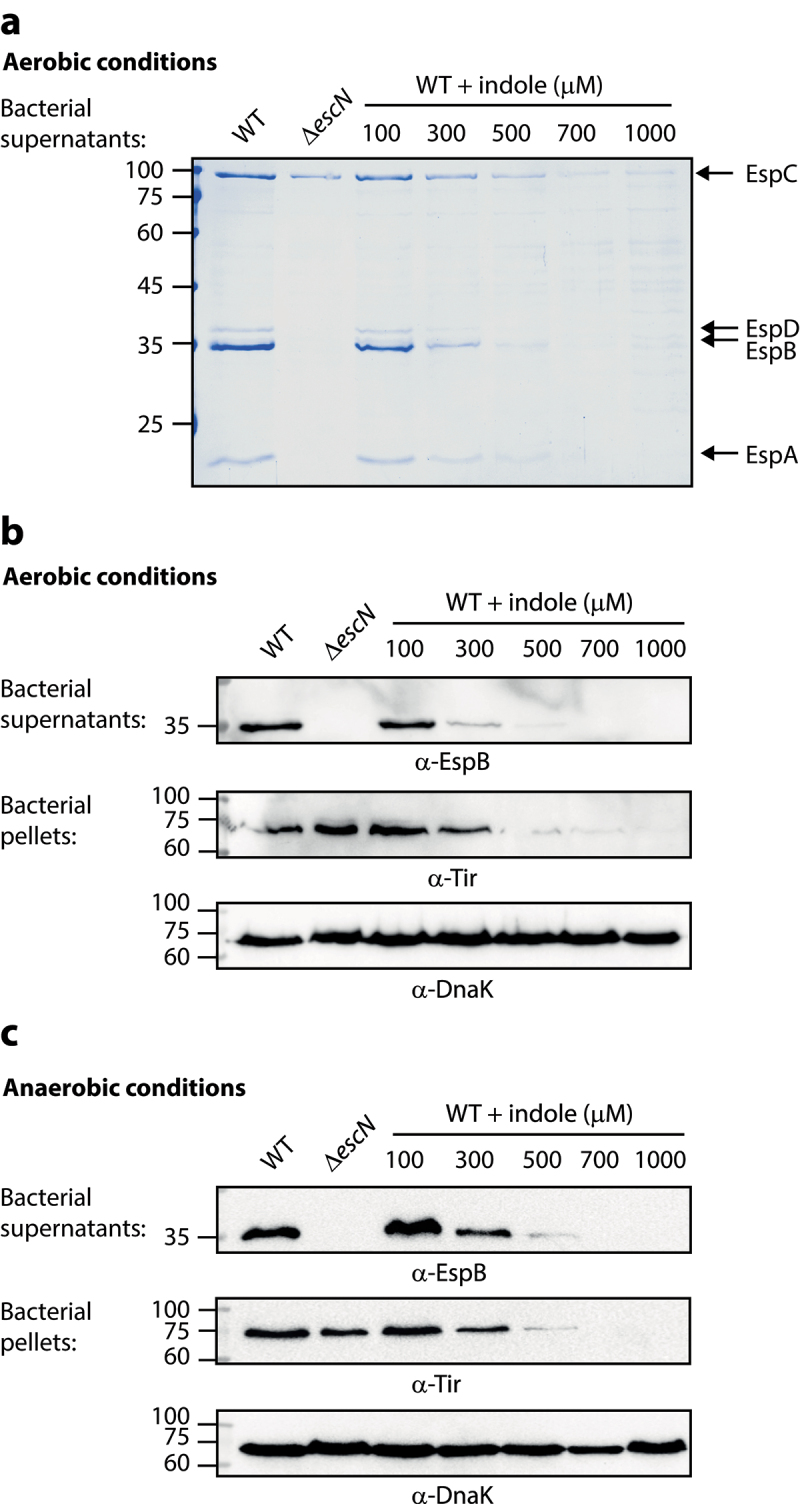
Indole inhibits EPEC T3SS activity in a dose-dependent manner. Wild type (WT) EPEC and *escN* null-mutant (Δ*escN*) EPEC were grown under optimal T3SS-inducing conditions aerobically (a-b) and anaerobically (c) for 6 h in the presence of various concentrations of indole. Bacterial supernatants and pellets were separated, normalized, and analyzed via 12% SDS-PAGE with Coomassie staining (a) or western blotting analyses performed using anti-EspB, anti-Tir, and anti-DnaK antibodies (b-c). DnaK levels were used to confirm equal levels of lysate loading in these different samples. In panel A, the T3SS-secreted translocators, EspA, EspB, and EspD, are marked on the right of the gel. The location of EspC, which is not secreted via the T3SS, is also marked.

Previous studies have reported that indole can alter various bacterial processes, as in the case of its ability to inhibit the virulence of enteric pathogens such as EHEC and *Citrobacter rodentium*.^25,31^ To examine the effect of indole on the T3SS activity, which is the primary virulence mechanism exhibited by EPEC, we grew WT EPEC under optimal T3SS-inducing conditions (DMEM, statically) in aerobic and anaerobic environments in the presence of different indole concentrations (Figure 1). As the physiological concentration of indole in the gastrointestinal tract of humans and mice has been suggested to be as high as 1 mM,^23,32,34–37^ we examined the effects of indole concentrations ranging from 100–1000 µM. T3SS activity was assessed by measuring the ability of EPEC to secrete T3SS translocators (EspA, EspB, and EspD) into culture supernatants. We observed efficient secretion of these translocators by WT EPEC, whereas no translocators were detected in the supernatants collected from the Δ*escN* null strain, which harbors a deletion of the T3SS ATPase gene (Figure 1). Analyses of the supernatants prepared from WT EPEC grown in the presence of indole concentrations at or above 300 μM exhibited reduced levels of secreted extracellular T3SS-associated proteins relative to DMSO vehicle control-treated samples (Figure 1a). To better monitor the effects of indole on T3SS activity, we analyzed the supernatants and bacterial pellets via western blotting using anti-EspB and anti-Tir antibodies, revealing that indole inhibited EspB secretion in a dose-dependent manner (Figure 1b and 1c). We also assessed the expression of the T3SS effector protein Tir, which should be retained within the bacterial cells at this stage, by analyzing whole-cell bacterial pellets. This analysis revealed that indole similarly inhibited Tir expression in a dose-dependent manner within the bacterial pellets (Figure 1b and 1c). DnaK levels were used to confirm equal levels of lysate loading in these different samples. To exclude the possibility that indole reduced bacterial virulence by inhibiting bacterial growth, we grew WT EPEC under optimal T3SS-inducing conditions in the presence (500 μM) or absence of indole and monitored optical density values over time, observing similar growth rates irrespective of the presence of indole (Fig. S1). Overall, these results indicated that physiological concentrations of indole inhibit EPEC T3SS secretion activity.

### Indole impairs the enhancement of EPEC virulence in response to V. cholerae growth under co-culture conditions.

**Figure 2.**
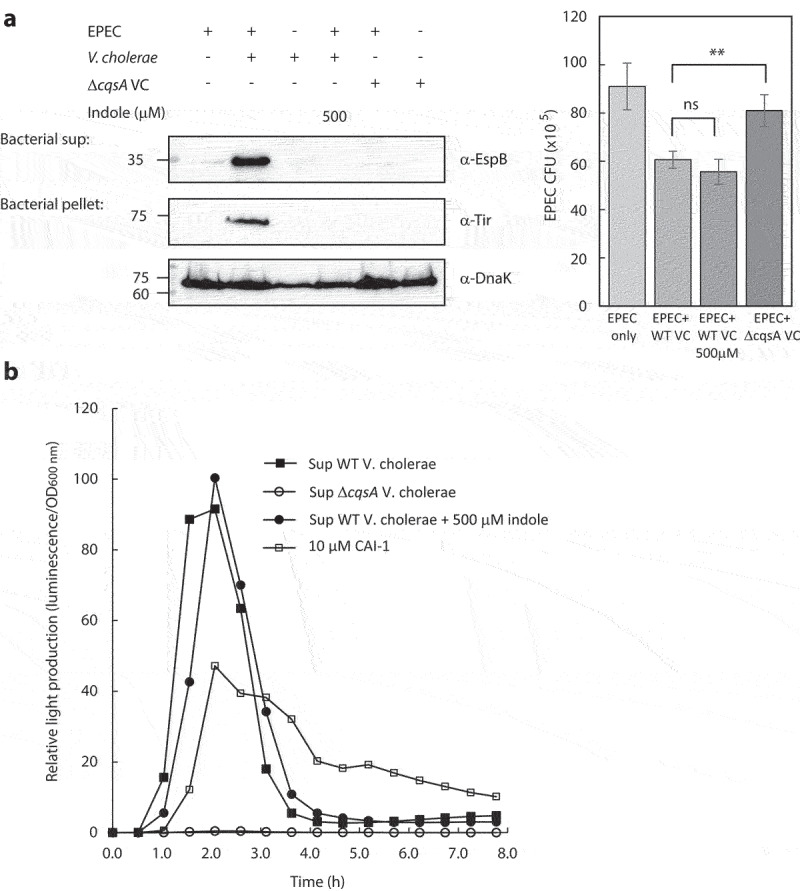
Indole interferes with the crosstalk between EPEC and *V. cholerae*. (a) Pure overnight cultures of EPEC and *V. cholerae* strains were sub-cultured in fresh 1:1 (v/v) DMEM: LB as single or mixed cultures. These cultures were grown in the presence or absence of indole under semi-optimal T3SS-inducing conditions for 6 h, and then the bacterial pellets and supernatants (bacterial sup) were separated, normalized, and analyzed. The secreted proteins were concentrated from collected supernatants and analyzed via 12% SDS-PAGE and western blotting using an anti-EspB antibody. The expression of the effector protein Tir, which should remain present primarily within the bacterial cytoplasm at this stage, was analyzed by subjecting the bacterial pellets to SDS-PAGE and western blotting using an anti-Tir antibody. Samples were also probed with anti-DnaK to confirm equal loading. Samples from the cultures were plated on LB plates containing chloramphenicol for EPEC growth. The plates were incubated overnight at 37°C, and bacterial colony-forming units (CFUs) were then counted. CFUs are averaged from three replicates of a representative experiment; error bars correspond to the standard error of the mean; **P < .005. ns indicates non significant. (b) Relative light production was used as means of assessing the levels of CAI-1 produced by WT *V. cholerae* in the absence or presence of indole (500 µM) and by Δ*cqsA V. cholerae* strain. Synthetic CAI-1 (10 µM) was used as a positive control. Data are averaged from three replicates of a representative experiment.

We previously reported that EPEC modulates its virulence in response to the size of *V. cholerae* populations by sensing and responding to elevated concentrations of CAI-1.^7^ To evaluate EPEC virulence when grown in co-culture with *V. cholerae*, we inoculated these bacterial strains into a 1:1 (v/v) mixture of DMEM and Luria-Bertani (LB) broth. This mixture corresponds to semi-optimal T3SS-inducing conditions, as it induces only partial T3SS activation and leaves room for an additional T3SS enhancement. As expected, we observed elevated EspB secretion and Tir expression levels in EPEC and *V. cholerae* co-culture sample relative to these levels in the pure EPEC culture sample (Figure 2a). To reaffirm that EPEC T3SS upregulation is CAI-1 dependent, we generated a *V. cholerae* Δ*cqsA* mutant strain in which the CAI-1 synthase gene *cqsA* had been deleted, and examined the ability of these bacteria to alter EPEC T3SS responses. EPEC co-cultured with the *V. cholerae* Δ*cqsA* mutant strain exhibited weak EspB secretion and no Tir expression, with these levels more closely resembling those for pure EPEC cultures (Figure 2a). These results further suggest that the upregulation of EPEC T3SS activity is CAI-1 dependent.

DnaK levels in the bacterial pellets showed equal sample loading. However, since the anti-DnaK antibody detects both EPEC and *V. cholerae*, we assessed EPEC numbers to confirm that the upregulated T3SS we observed was not due to a higher bacterial count of EPEC. For that purpose, we plated the pure and co-cultures on selective media to determine the number of EPEC colony forming units (CFUs). We observed that EPEC showed the highest CFUs when grown as a pure culture, and had a ~ 30% reduction in CFUs when grown in co-culture with WT *V. cholerae* (with or without indole) and a milder reduction (~10%) when grown in co-culture with *V. cholerae* Δ*cqsA* mutant (Figure 2a). These results confirmed that the T3SS upregulation we observed for EPEC co-culture with WT *V. cholerae* was not due to a higher EPEC count relative to the pure EPEC culture. In addition, the CFUs of the co-culture with indole, which had similar CFUs as the co-culture without indole, and of the co-culture with *V. cholerae* Δ*cqsA* mutant, which was higher than co-culture with WT *V. cholerae*, confirmed that the neutralization of T3SS upregulation by indole and the lack of upregulation by *V. cholerae* Δ*cqsA* mutant were not due to EPEC counts. In parallel, we examined the CFUs of *V. cholerae* in the pure and co-culture samples. We observed a mild variability between the samples (Fig. S2A), which could not account for the effect on T3SS activity, except for the co-culture of EPEC and *V. cholerae* Δ*cqsA*, which showed a significant decrease in *V. cholerae* CFUs. To confirm that the lack of T3SS upregulation in this co-culture was not due to the low count of Δ*cqsA V. cholerae*, we co-cultured EPEC with a higher inoculum of *V. cholerae* Δ*cqsA* to obtain similar CFUs as that of the co-culture with WT *V. cholerae*. We observed that this co-culture of EPEC and *V. cholerae* Δ*cqsA* exhibited similar T3SS activity (EspB secretion and Tir expression) as the pure EPEC culture (Fig. S2A), thus excluding the possibility that the lack of T3SS upregulation is due to the low bacterial count.

To characterize EPEC T3SS responses in the presence of *V. cholerae* under conditions that better simulate the human gastrointestinal tract, we performed co-culture experiments in the presence of indole. The addition of indole (500 µM) to the co-culture of EPEC and WT *V. cholerae* completely abolished EspB secretion and Tir expression (Figure 2a), indicating that indole impairs the ability of EPEC respond to *V. cholerae*’s presence by upregulating its T3SS accordingly. Since EPEC and *V. cholerae* are known to endogenously produce indole,^26,38^ we measured the indole concentrations in the bacterial cultures grown under semi-optimal T3SS-inducing conditions. We found that EPEC produces ~150 µM indole while *V. cholerae* produces neglectable indole levels, therefore not reaching adequate indole concentrations to inhibit EPEC T3SS activity. To validate that the inability of EPEC to respond to *V. cholerae* presence was not due to the effects of indole on CAI-1 production, we assessed the CAI-1 concentrations produced by *V. cholerae* grown in the presence or absence of indole using the MM920 *V. cholerae* reporter strain, which contains the *V. harveyi lux*CDABE luciferase operon that is activated by CAI-1.^39^ We incubated this reporter strain with supernatants prepared from WT *V. cholerae* grown in the presence (500 µM) or absence of indole and measured light production over time. Supernatants prepared from the Δ*cqsA V. cholerae* strain and synthetic CAI-1 were used as negative and positive controls, respectively. While no light production was observed from the reporter strain grown in presence of Δ*cqsA V. cholerae* supernatant, a strong signal was detected when the reporter strain was grown in the presence of WT *V. cholerae* supernatant irrespective of the presence or absence of indole (Figure 2b). These results indicate that indole did not alter CAI-1 production. To further exclude the possibility that indole reduced EPEC T3SS responses due to its effect on *V. cholerae* growth, we compared bacterial growth rates in the presence (500 μM) or absence of indole by monitoring the optical density over time. We observed similar growth rates regardless of indole presence (Fig. S2B). Our results thus suggested that indole impairs the ability of EPEC to upregulate its T3SS activity in response to the presence of *V. cholerae*.

### Indole interferes with EPEC responses to CAI-1 at high micromolar concentrations

**Figure 3.**
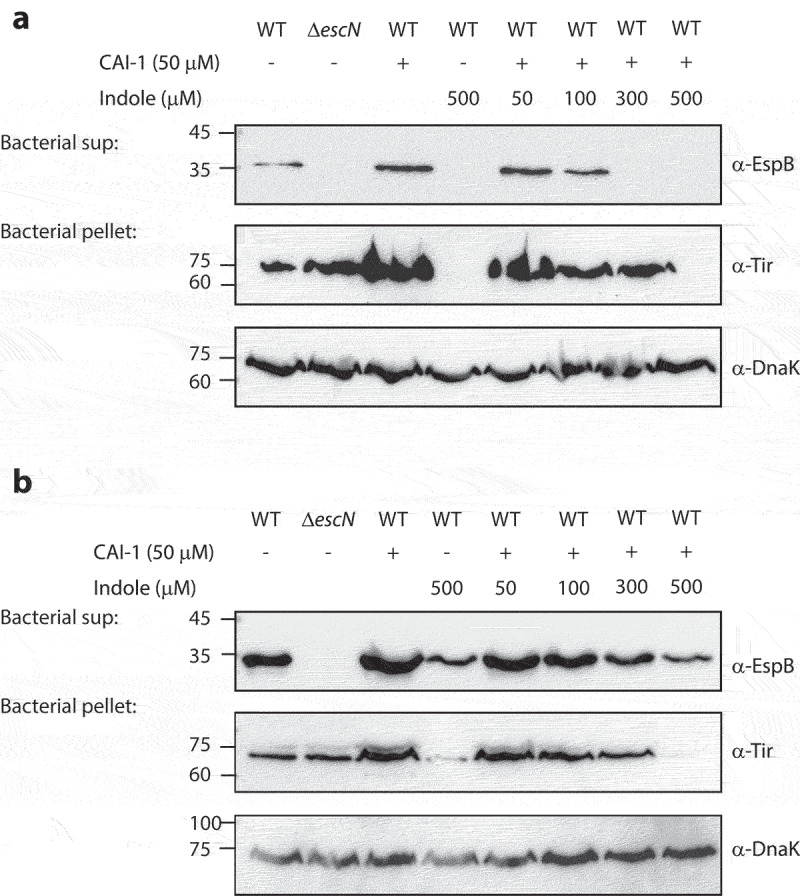
Indole competes with CAI-1 to influence EPEC T3SS activation. Wild type (WT) EPEC and *escN* null-mutant (Δ*escN*) EPEC were grown for 6 h under semi-optimal (a) or optimal (b) T3SS-inducing conditions in the presence of CAI-1 (50 µM) and various concentrations of indole (50–500 µM). The secreted proteins were concentrated from bacterial culture supernatants (bacterial sup) and analyzed via 12% SDS-PAGE and western blotting using an anti-EspB antibody. The expression of the effector protein Tir, was analyzed by subjecting the bacterial pellets to SDS-PAGE and western blotting using an anti-Tir antibody. Samples were also probed with anti-DnaK to confirm the equal loading of lysates.

To study the interplay between indole, CAI-1, and EPEC T3SS responses, we examined T3SS activity levels at various synthetic CAI-1/indole molar ratios. To more sensitively detect the enhancement of the T3SS activity, we cultured EPEC under semi-optimal T3SS-inducing conditions that do not induce full T3SS activation, providing the opportunity for further T3SS upregulation. These bacterial cultures were then separated into supernatants and bacterial pellet samples and were analyzed to detect EspB secretion (supernatants) and Tir expression (bacterial pellets). As expected, we observed elevated levels of EspB secretion and Tir expression when WT EPEC were cultured in the presence of CAI-1 (50 µM) relative to DMSO control (Figure 3a). Moreover, a similar elevation was observed for EPEC grown in the presence of CAI-1 and indole at a 1:1 molar ratio (Figure 3a). However, at higher indole concentrations, the T3SS-upregulating effects of CAI-1 were curtailed in a dose-dependent manner. WT EPEC samples grown in the presence of a 1:10 ratio of CAI-1 and indole exhibited the complete elimination of EspB secretion and Tir expression (Figure 3a). DnaK levels within the bacterial pellets confirmed similar sample loading for these analyses.

To better determine the antagonistic effect of indole on CAI-1 responses, we cultured WT EPEC under optimal T3SS-inducing conditions that promote a maximal T3SS response. Under these conditions, we found that indole promoted a similar dose-dependent inhibition of EspB secretion and Tir expression levels, with a maximal effect at a 1:10 CAI-1/indole ratio (Figure 3b). Furthermore, although residual EspB secretion and Tir expression were observed in the presence of indole (500 µM), no upregulation was observed in the presence of CAI-1 (Figure 3b). These results suggested that indole may not only inhibits T3SS per se but also, at high micromolar concentrations, neutralizes the ability of CAI-1 to upregulate EPEC T3SS activity either by acting as a general inhibitor, which overwrites the activity of various inducers, or by masking pathogens’ communication. To confirm that the effect we observed represents the physiological concentration of CAI-1 produced by *V. cholerae*, we added *V. cholerae* supernatant in combination with various indole concentrations (Fig. S3). We observed similar effect on EPEC T3SS activity as we observed for the synthetic CAI-1 (Fig. S3), thus suggesting this effect is biologically relevant.

### Indole inhibits the CAI-1-induced upregulation of EPEC T3SS genes

**Figure 4.**
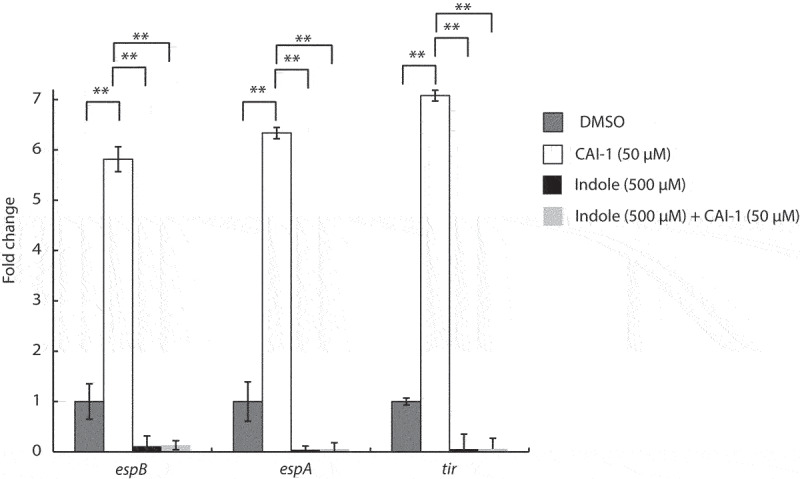
Indole suppresses the upregulation of EPEC T3SS genes induced by CAI-1. WT EPEC was grown for 2 h under semi-optimal T3SS-inducing conditions in the presence of 0.5% (v/v) DMSO (dark gray bars), CAI-1 (white bars), indole (black bars), or both CAI-1 and indole (light gray bars). mRNA levels for the T3SS genes, *espB, espA*, and *tir*, were measured via qRT-PCR. mRNA levels are presented relative to those of WT EPEC grown in the presence of DMSO (dark gray bars). Data are averaged from three replicates of a representative experiment; error bars correspond to the standard error of the mean; **P < .005.

To examine whether indole affects the transcription of T3SS genes, we cultured WT EPEC under semi-optimal T3SS-inducing conditions. We then added CAI-1 alone or together with indole at a 1:10 molar ratio and evaluated the transcription of three representative LEE genes; *tir* – the first translocated effector encoded on the LEE5 operon, and two T3SS translocators, *espA* and *espB*, encoded on the LEE4 operon. As observed previously, we detected significantly elevated levels of these T3SS transcripts when bacteria were cultured in the presence of CAI-1 as compared to DMSO alone (Figure 4). However, bacteria grown in the presence of CAI-1 and indole at a 1:10 molar ratio exhibited almost complete abrogation of the transcription of all three genes (Figure 4). These results indicated that indole reduces T3SS activity by downregulating the transcription of T3SS genes, and this reduction is not relieved even under CAI-1-inducing conditions.

### Microbiome-derived indole impairs the ability of EPEC to sense and respond to the presence of V. cholerae

**Figure 5.**
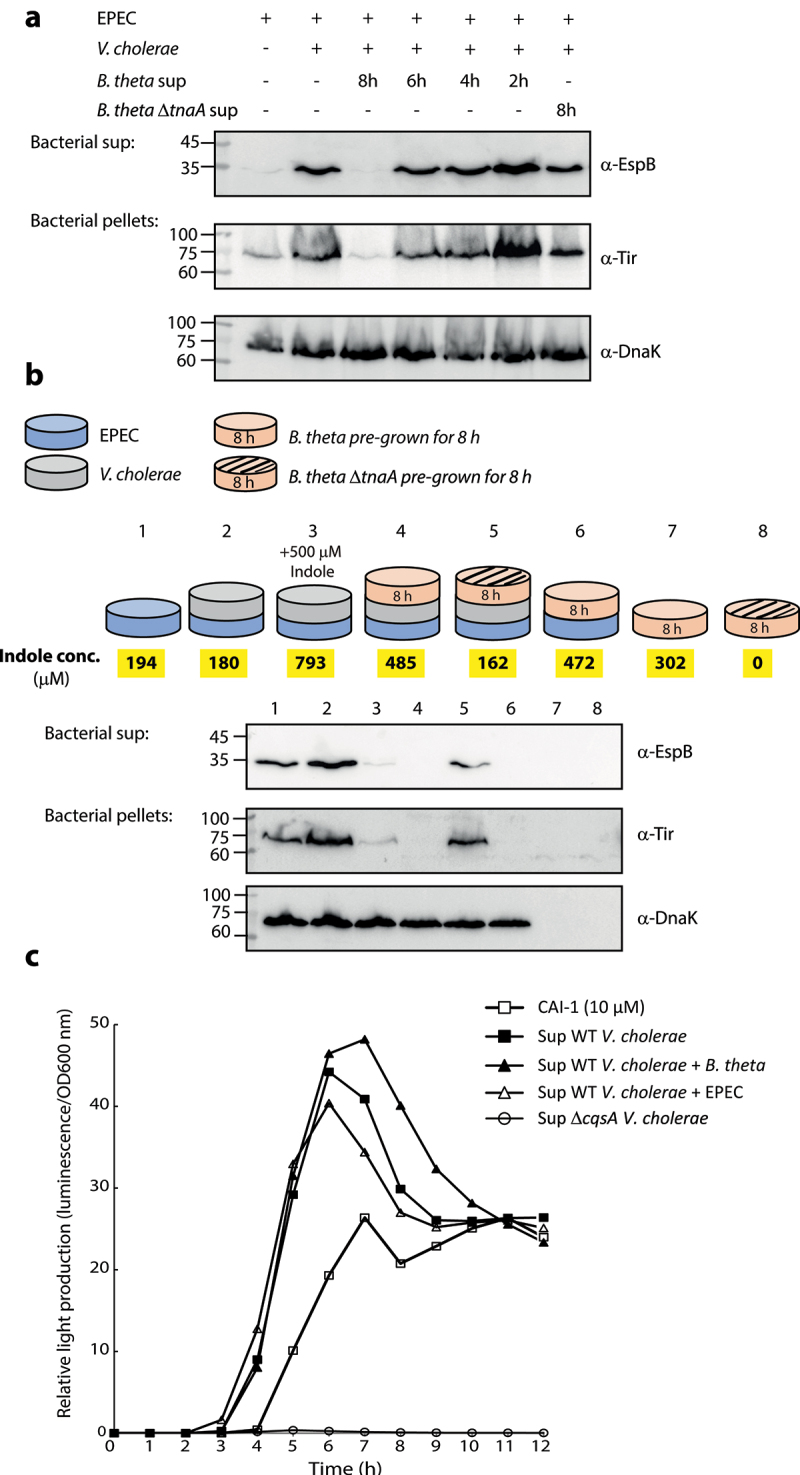
*B. thetaiotaomicron*-derived indole inhibits the enhancement of EPEC T3SS activity upon co-culture with *V. cholerae*. (a) Supernatants of WT *B. thetaiotaomicron* (*B. theta*) grown for 2, 4, 6 and 8 h and Δ*tnaA B. thetaiotaomicron* grown for 8 h were added to co-cultures of EPEC and *V. cholerae* grown in DMEM: BHI mixture aerobically, for 6 h. The bacterial pellets and supernatants (bacterial sup) were separated, normalized, and analyzed. The secreted proteins were concentrated from culture supernatants and analyzed via 12% SDS-PAGE and western blotting using an anti-EspB antibody. The expression of the effector protein Tir, was analyzed by subjecting the bacterial pellets to SDS-PAGE and western blotting using an anti-Tir antibody. Samples were also probed with anti-DnaK to confirm equal loading. (b) A schematic overview of the bacterial combinations and indole supplementation used for this experiment. WT EPEC was sub-cultured in a mixture of 1:1 (v/v) DMEM: BHI medium as pure culture (sample 1), a co-culture with *V. cholerae* (sample 2), a co-cultured with *B. thetaiotaomicron* (sample 6), or a tri-culture with *V. cholerae* and *B. thetaiotaomicron* (sample 4 with WT *B. thetaiotaomicron* and sample 5 with Δ*tnaA B. thetaiotaomicron*). *B. thetaiotaomicron* were pre-grown for 8 h before EPEC and *V. cholerae* were added. One of the EPEC and *V. cholerae* co-cultures was supplemented with 500 µM indole (sample 3). Pure cultures of WT and Δ*tnaA B. thetaiotaomicron* were used as negative controls (samples 7 and 8). All cultures were grown anaerobically for 6 h and their indole concentrations were determined (average values are highlighted in yellow). The bacterial pellets and supernatants (bacterial sup) were separated, normalized, and analyzed as described in panel A. (c) Relative light production was used to assess the levels of CAI-1 produced by WT *V. cholerae* when grown alone or in co-culture with either *B. thetaiotaomicron* (*B. theta*) or EPEC. Synthetic CAI-1 (10 µM) and the supernatant of Δ*cqsA V. cholerae* strain, which cannot produce CAI-1, were used as positive and negative controls, respectively. Data are averaged from three replicates of a representative experiment.

To examine the effect of bacteria-produced indole on the communication between EPEC and *V. cholerae*, we employed the commensal bacteria *Bacteroides thetaiotaomicron*, a highly abundant gut bacteria that naturally converts tryptophan into indole.^25,26,40^ Since *B. thetaiotaomicron* is an obligate anaerobe, we grew it separately under anaerobic conditions, collected its supernatants at various time points, and added them to EPEC and *V. cholerae* co-culture (grown aerobically for 6 h). We then separated the cultures into supernatants and bacterial pellet samples and analyzed EspB secretion (supernatants) and Tir expression (bacterial pellets). We observed a clear inhibitory effect on EspB secretion and Tir expression when adding supernatant of 8 h growth of *B. thetaiotaomicron*, while supernatants of shorter culture-times had mild to no inhibitory effect (Figure 5a). Importantly, the addition of the supernatant of *B. thetaiotaomicron* Δ*tnaA* strain, which cannot produce indole, after 8 h growth, showed negligible inhibitory effect on T3SS activity, compared to the supernatant of WT *B. thetaiotaomicron* (Figure 5a).

Next, we performed a multi-bacteria culture assay that more closely resembles the intestinal environment. *B. thetaiotaomicron* was sub-cultured together with EPEC and *V. cholerae* under anaerobic conditions in a 1:1 (v/v) mixture of DMEM and BHI. These semi-optimal T3SS-inducing conditions were conducive to the growth of all three bacterial strains. We then compared the T3SS activity of EPEC grown under tri-culture conditions, either with WT or Δ*tnaA B. thetaiotaomicron*, to that of EPEC grown as a pure culture or co-cultured with either *B. thetaiotaomicron* or *V. cholerae*, as shown in Figure 5b. *B. thetaiotaomicron* pure cultures served as negative controls.

As expected, the co-culture of EPEC and *V. cholerae* induced higher levels of T3SS secretion activity compared to that of EPEC pure culture (Figure 5b). However, the tri-culture of EPEC, *V. cholerae*, and WT *B. thetaiotaomicron* completely abolished EPEC T3SS activity and resembled the EspB/Tir levels detected for the sample of EPEC and *V. cholerae* co-culture grown in the presence of 500 µM indole (Figure 5b). In addition, the co-culture of EPEC and WT *B. thetaiotaomicron* eliminated EspB secretion and Tir expression (Figure 5b). Remarkably, tri-culture of Δ*tnaA B. thetaiotaomicron*, EPEC, and *V. cholerae* was associated with higher levels of EspB secretion and Tir expression compared to the tri-culture with WT *B. thetaiotaomicron* (Figure 5b). This suggested that indole is involved, even if not exclusively, in alternating the T3SS response of EPEC. DnaK levels in the prepared bacterial pellets confirmed equal sample loading for these analyses. Unsurprisingly, the *B. thetaiotaomicron* pure cultures were negative for DnaK expression as the utilized anti-DnaK antibody reacts primarily with DnaK proteins derived from *E. coli* or closely related bacteria, such as *V. cholerae*. Measurement of indole concentrations within the cultures (Figure 5b, highlighted in yellow) revealed that the T3SS inhibitory effect is observed in cultures with indole concentrations of ~500 µM and higher. These results further supported our previous observations by demonstrating that microbiome-derived indole inhibits EPEC T3SS activity, even under highly inducible conditions.

To rule out the possibility that *B. thetaiotaomicron* affects CAI-1 production of *V. cholerae*, we co-cultured the strains, collected the supernatant, and assessed the CAI-1 concentration using the MM920 *V. cholerae* reporter strain.^39^ Supernatants prepared from the Δ*cqsA V. cholerae* strain and synthetic CAI-1 (10 µM) were used as negative and positive controls, respectively. While no light production was observed from the reporter strain grown in the presence of Δ*cqsA V. cholerae* supernatant, a strong signal was detected when the reporter strain was grown in the presence of supernatants of WT *V. cholerae* grown alone or co-cultured with EPEC or *B. thetaiotaomicron* (Figure 5c). These results indicated that *B. thetaiotaomicron* did not alter CAI-1 production. To exclude the possibility that *B. thetaiotaomicron* cultures inhibited EPEC and *V. cholerae* growth, we compared the bacterial counts following co-culture and tri-culture growth by plating these cells on a selective medium. We found that EPEC and *V. cholerae* counts were unaffected by the presence of *B. thetaiotaomicron*, which was viable under these growth conditions (Fig. S4). Therefore, we conclude that microbiome-derived indole can interfere with the synergy between EPEC and *V. cholerae* pathogens.

### Indole inhibits the ability of EPEC to translocate effector proteins into host cells

**Figure 6.**
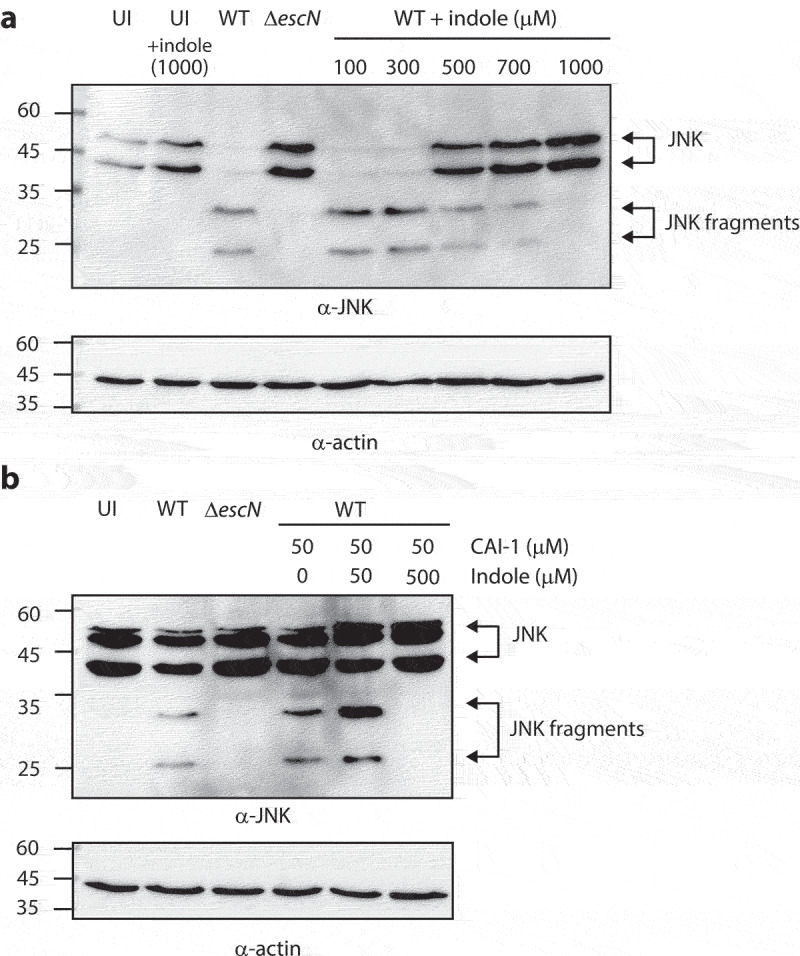
Indole reduces the ability of EPEC to translocate NleD into host cells, even in the presence of CAI-1. (a) HeLa cells were infected with WT and Δ*escN* EPEC strains grown under optimal T3SS-inducing conditions in the presence of various indole concentrations (100–1000 µM) for 3 h. Cells were washed, and their proteins were extracted and subjected to western blotting analysis using anti-JNK and anti-actin (loading control) antibodies. JNK and its degradation fragments are indicated to the right of the gel. (b) Western blotting analysis of JNK degradation patterns following HeLa infection with WT and Δ*escN* EPEC strains grown under semi-optimal T3SS-inducing conditions in the absence or presence of CAI-1 (50 µM) and indole (50 or 500 µM) for 2 h.

To further evaluate the effects of indole on EPEC virulence, we utilized a bacterial infection model that examines the ability of WT EPEC to infect HeLa cells and promote the translocation of effectors into host cells. We infected HeLa cells with EPEC strains grown under optimal T3SS-inducing conditions, in the presence of varying concentrations of indole, and monitored the cleavage of host cell-derived c-Jun N-terminal kinase (JNK), which is degraded by a translocated effector protein NleD.^41^ As expected, HeLa cells infected with WT EPEC cultures exhibited JNK degradation, in contrast to the uninfected HeLa sample and the sample infected with Δ*escN* EPEC strain (Figure 6a). However, HeLa cells infected with WT EPEC cultures pre-incubated with indole at a concentration at or above 500 μM exhibited reduced JNK degradation and higher levels of full-length JNK (Figure 6a). These results suggested that indole inhibits the virulence of EPEC by interfering with the ability of its T3SS to translocate effector proteins into host cells.

As we previously reported that CAI-1 enhances the ability of EPEC to infect HeLa cells and to translocate effectors into host cells,^7^ we further examined whether this enhancement was altered in the presence of indole. To that end, we monitored the cleavage patterns of JNK in HeLa cells infected with EPEC strains grown under semi-optimal T3SS conditions (to ensure that there was an opportunity for enhanced virulence) incubated with both CAI-1 and indole at a 1:1 or 1:10 molar ratio. As expected, HeLa cells infected with WT EPEC in the presence of CAI-1 exhibited higher levels of JNK degradation as compared to those infected with WT EPEC alone. While high JNK degradation levels were still observed for the sample infected with WT EPEC incubated with CAI-1 and indole at a 1:1 ratio, complete inhibition of JNK degradation was detected for EPEC incubated with CAI-1 and indole at a 1:10 ratio (Figure 6a). These results suggested that indole suppresses the enhancement of EPEC virulence induced by CAI-1.

## Discussion

The gastrointestinal microbiome plays a critical role in human health, in part because it provides colonization resistance against pathogenic bacteria.^42,43^ This is achieved owing to the ability of the microbiome to produce various metabolites (e.g., essential vitamins, carbohydrates, peptides, and lipopolysaccharides) that enhance host immunity.^24,42–45^ In addition, these microbiome-derived metabolites can directly inhibit pathogen virulence mechanisms and therefore protect against bacterial infections. For example, *Bacteroides thuringiensis* and commensal *E. coli* synthesize peptides known as bacteriocins that inhibit the virulence of *Enterococcus faecalis, Klebsiella pneumonia, Salmonella*, and EHEC.^46–49^

The ability of indole, a microbiome-derived metabolite, to directly inhibit bacterial virulence and reduce the infection capabilities of several enteric pathogens has previously been demonstrated.^25,33,50^ In this study, we extended this finding to EPEC and found that indole directly inhibits EPEC T3SS secretion activity, at physiological concentrations under aerobic and anaerobic conditions (Fig. 1 and 5). These results are in keeping with a previous study that reported that indole and its derivatives alter the motility, biofilm formation, and Shiga toxin production activities of various pathogenic *E. coli* strains.^51^

In this study, we examined not only whether indole was able to inhibit bacterial virulence, but also whether it could interfere with the bacterial communication related to virulence enhancement or overwrite synergy between pathogens. For that purpose, we used an EPEC and *V. cholerae* co-infection model, having previously demonstrated that these two pathogens time their virulence to reduce their competition and coordinate their infectious processes.^7^ We have previously suggested that this communication is mediated by CAI-1, the primary QS molecule produced by *V. cholerae*. Here, we confirmed this model by generating a *V. cholerae* mutant strain in which the CAI-1 synthase gene, *cqsA*, had been deleted such that these bacteria were deficient for CAI-1 production. Unlike co-culture with WT *V. cholerae*, co-culture of EPEC and *V. cholerae* Δ*cqsA* did not induce the upregulation of EPEC T3SS activity (Figure 2), thus suggesting that this bacterial communication is mediated through CAI-1. Nevertheless, since the Δ*cqsA* mutant strain is QS defective (locked at low cell density state), it produces and secretes different extracellular metabolites than the WT strain.^52^ These might also play roles in changing EPEC virulence behavior.

Using this inter-bacterial communication system, we discovered that the addition of indole or *B. thetaiotaomicron*, which produce indole, to the EPEC and *V. cholerae* co-culture model system not only inhibited EPEC T3SS activity but also disrupted the communication between these pathogens, which altogether result in the ablation of EPEC T3SS activity upregulation (Figure 2 and Figure 5). This effect is solely related to the concentration of indole and not to its origin. Therefore, indole production by EPEC and *V. cholerae*, which has been reported to occur primarily during their stationary growth phase,^26^ can eventually affect bacterial communication but is less relevant to the experimental setup presented in this study. We observed that the ability of CAI-1 to upregulate EPEC virulence was neutralized in an indole-dependent manner, primarily at higher indole concentrations (Figure 3 and Figure 5). This novel finding provides a possible explanation for the variability found among individuals with respect to their susceptibility to bacterial infections. This suggests that differences in microbiome composition can account for variations in gastrointestinal indole concentrations, which in turn can adjust virulence and alter the communication between gut pathogens, ultimately supporting or interfering with the process of bacterial infection.

This finding is in line with previous studies demonstrating a link between microbiome composition and certain gut-associated diseases such as inflammatory bowel disease, obesity, type 2 diabetes, and even cancer.^53,54^ The deliberate alteration of the microbiota may thus offer potential as a therapeutic tool.^54^ Given that our results demonstrate that microbiome-derived indole was sufficient to inhibit virulence, either by acting as a general LEE inhibitor or by interfering with the pathogen communication that disrupt the ability of pathogens to coordinate infections, it is logical to assume that the enrichment of indole-producing bacterial species within the microbiome will provide more robust colonization resistance, particularly against simultaneous infection with multiple enteric pathogens.^1–3^ In addition, to promote indole production by these strains, the consumption of a protein-rich diet should be encouraged, as indole is produced via the metabolism of tryptophan.^55,56^ A protein-rich diet may thus aid in preventing bacterial infections. Furthermore, indole has the potential to be developed into a postbiotic supplement, which is defined as a bioactive compound naturally produced by the gut microbiome that has been shown to improve human health.

The ability of bacterial species to monitor their population size as well as the population size of potentially competing species is vital for the coordination of group behavior that is required for the infection process and survival within a given host. Interference with this process can therefore be a potent means of combatting bacterial infection. An example of such interference was previously described by Xavier and Bassler, who found that *E. coli* interfere with *V. cholerae* and *V. harveyi* QS signaling by actively internalizing their QS molecule, AI-2.^57^ This mechanism results in *Vibrio* species miscalculating their population size, thereby interfering with the ability of these bacteria to properly respond to changes in their cell population density, with these responses often being crucial for successful bacterial-host relationships. In this study, we described an additional mechanism whereby a microbiome-derived metabolite can interfere with bacterial communication. The observation that gastrointestinal commensal species produce a specific component that plays an important role in promoting gut health may be representative of a broader phenomenon, potentially highlighting a novel approach to combatting infectious diseases. As such, further studies of CAI-1 and indole signaling are warranted, including efforts to define the EPEC CAI-1 receptor and to determine whether indole acts as an antagonist of this receptor. A more detailed understanding of the direct and indirect effects of indole on bacterial virulence will aid in the development of novel anti-virulence therapeutics.

## Materials and methods

### Bacterial strains

The wild-type (WT) enteropathogenic *E. coli* (EPEC) O127:H6 strain E2348/69 (streptomycin-resistant) and the Δ*escN* null strain (Table 1) were grown at 37°C in Luria-Bertani (LB) broth (Sigma) supplemented with the appropriate antibiotics unless otherwise indicated. *E. coli* (EPEC) O127:H6 strain E2348/69 carrying pACYC184 empty vector (Table 1) was grown in LB broth supplemented with chloramphenicol. *V. cholerae* O1 In ET-122 (+) strains (WT, Δ*cqsA*, and the MM920 reporter strain – Table 1) were grown at 30°C in LB broth supplemented with appropriate antibiotics. *B. thetaiotaomicron* (Table 1) was grown at 37°C in Brain Heart Infusion (BHI, Sigma) broth under static, anaerobic conditions. The following antibiotics were used for this study: streptomycin (50 µg/mL), carbenicillin (100 µg/mL), chloramphenicol (35 µg/mL), and tetracycline (12.5 µg/mL).

**Table 1.** Strains used in this study.

Strain	Description	Reference
WT EPEC	EPEC strain E2348/69, streptomycin resistant	^58^
WT EPEC + pACYC184	EPEC strain E2348/69, streptomycin and chloramphenicol resistant	This study
EPEC Δ*escN*	Nonpolar deletion of *escN*	^59^
*Vibrio cholerae*	*V. cholerae* biotype El-Tor serotype Inaba O1 In ET-122 (+)	^60^
*Vibrio cholerae* Δ*cqsA*	Nonpolar deletion of *cqsA in V. cholerae*	This study
*V. cholerae* MM920 reporter strain	*V. cholerae* biotype EI-Tor serotype Δ*cqsA*Δ*luxQ* with pBB1 cosmid containing the *V. harveyi* luxCDABE operon (tetracycline resistant)	^50^
WT *Bacteroides thetaiotaomicron*	*B. thetaiotaomicron* VPI-5482 Δ*tdk* (nalidixic acid resistant)	^61^
*B. thetaiotaomicron* Δ*tnaA*	*B. thetaiotaomicron* VPI-5482 Δ*tdk*Δ*tnaA*	^40^
*E. coli* SM10λpir	For bacterial conjugation	^62^

### *Construction of the null Δ*cqsA V. cholerae mutant strain

Nonpolar deletion of *cqsA* in *V. cholerae* O1 In ET-122 (+) was achieved by using the *sacB*-based allelic exchange method.^63^ Briefly, two PCR fragments corresponding to the flanking regions of *cqsA* (0.9 and 1.18 kb, from the 5’ and 3’ of *cqsA*, respectively) were generated with the corresponding primer pairs cqsA_UF/cqsA_UR and cqsA_DF/ cqsA_DR (Table 2). The fragments were then annealed using the cqsA_UF/cqsA_DR primer pair and cloned into the pRE112 suicide vector. The resultant pRE112 plasmid contained the *cqsA* flanking regions, with 94% of *cqsA* having been deleted. The plasmid was then transformed into the *E. coli* SM10λpir conjugative strain to be introduced into WT *V. cholerae*.^62^ After a sucrose selection process, *V. cholerae* colonies that were resistant to sucrose and susceptible to chloramphenicol were screened for the deletion of *cqsA* by PCR. The deletion of the *cqsA* gene was then confirmed by sequencing (Table 2).

**Table 2.** Sequences of primers used in this study.

Null mutant	Primer name	Primer sequence	Reference
*V.cholerae ΔcqsA*	cqsA_UF	GAGCTCGATATCGCATGCTGCCCCCTTCACAAGC	This study
	cqsA_UR	CACCGTAGTTGACCGCATCATCAGGAAGTTGAGGCTTG	
	cqsA_DF	CAAGCCTCAACTTCCTGATGATGCGGTCAACTACGGTG	
	cqsA_DR	CAAGCTTCTTCTAGAGGTACCCGCAGGGAGAACTACTGC	
Gene	Primer name	Primer sequence	Reference
*Rpoa*	rpoA_qPCR_F rpoA_qPCR_R	GGCGCTCATCTTCTTCCGAAT CGCGGTCGTGGTTATGTG	^64^
*espB*	EspB_qPCR_F EspB_qPCR_R	GGCTCTTTTGCTGCCATTAATAGC TCTGCTGCATCTGCAATACC	^7^
*espA*	EspA_qPCR_F EspA_qPCR_R	GTGCGAATGCGAGTACTTCGAC TTGCAGCCTGAAAAACACCGAGT	^7^
*Tir*	Tir_qPCR_F Tir_qPCR_R	GGACCCTCTGCATTTCGTGTTG GTCCCCCGGTAAAAACAAATCTG	^7^

### Type III secretion (T3S) assay

T3S assays were performed as previously described.^65,66^ Briefly, WT EPEC and Δ*escN* strains were grown overnight at 37°C in LB broth with appropriate antibiotics. The overnight cultures were diluted 1:40 into either pre-warmed complete high glucose Dulbecco’s modified Eagle’s medium (DMEM, Biological Industries), referred to as optimal T3SS-inducing medium, or a 1:1 (v/v) DMEM:plain LB medium, referred to as semi-optimal T3SS-inducing medium, with this media being supplemented with DMSO, CAI-1 (50 μM), or indole (100–1000 μM). These cultures were grown for 6 h at 37°C under aerobic conditions (in a tissue culture incubator with 5% CO_2_) or anaerobic conditions (in a DonWhitley A35 anaerobic workstation, with a gas mixture of 5% H_2_, 10% CO_2_, and 85% N_2_). The optical density at 600 nm (OD_600_) of these cultures (WT EPEC and Δ*escN* strains) was measured before the cultures were centrifuged at 20,000 × g for 5 min to separate the bacterial pellets, which were dissolved in SDS-PAGE sample buffer, from the culture supernatants. The supernatants were filtered through a 0.22 μm low-protein-binding filter, normalized according to the bacterial OD_600_, and the secreted proteins present therein were precipitated with 10% (v/v) trichloroacetic acid (TCA) overnight at 4°C. The samples were then centrifuged at 18,000 × g for 30 min at 4°C, and secreted protein precipitates were dissolved in SDS-PAGE sample buffer, with the residual TCA being neutralized using saturated Tris. Samples were then analyzed on SDS-PAGE gels with Coomassie Blue staining (InstantBlue, Abcam) or via western blotting.

### Bacterial co- and multi-cultures

EPEC and *V. cholerae* cultures were grown separately overnight at 37°C (EPEC) or 30°C (*V. cholerae*) in LB broth. *B. thetaiotaomicron* cultures were grown anaerobically overnight at 37°C in BHI broth. For co-culture assays performed under aerobic conditions, EPEC and *V. cholerae* overnight cultures were diluted 1:40 into semi-optimal T3SS-inducing medium (1:1 [v/v] DMEM: LB) and grown together in a tissue culture incubator (with 5% CO_2_) statically for 6 h, either alone or in the presence of indole (500 µM – Arcos Organics). For co- and tri-culture assays performed under anaerobic conditions, *B. thetaiotaomicron* overnight cultures (WT and Δ*tnaA*) were diluted 1:16 into 1:1 (v/v) DMEM: BHI medium and grown for 8 h. Then, WT EPEC alone or with WT *V. cholerae* (each diluted 1:40) were added into *B. thetaiotaomicron* growth medium, while samples of pure cultures were left untreated, and cultured for an additional 6 h under anaerobic conditions. In addition, samples of EPEC only and co-cultures of EPEC and *V. cholerae* with 500 µM indole were added to 1:1 (v/v) DMEM: BHI medium and cultured for 6 h under anaerobic conditions. These cultures were then separated into bacterial supernatants and pellets and processed as described above for the T3S assay.

### Bioluminescence (LuxR) assay

The presence of CAI-1 in the culture media was determined by assessing light production mediated by the *V. cholerae* Δ*cqsA*Δ*luxP* reporter strain harboring the luxCDABE operon (MM920). *V. cholerae* MM920, WT *V. cholerae*, and *V. cholerae* Δ*cqsA* were grown overnight at 30°C in LB broth. The reporter strain was diluted 1:20 into fresh LB medium in white 96-well clear-bottom plates and was mixed with the supernatants of WT *V. cholerae*, Δ*cqsA* null strain, co-cultures of WT *V. cholerae* with EPEC, co-cultures of WT *V. cholerae* with *B. thetaiotaomicron*, or WT *V. cholerae* grown in the presence of 500 µM indole. Plates were then incubated at 30°C with aeration, and light production and OD_600_ values were measured every 30 min (TECAN Infinite 200Pro). Plain LB and 10 µM CAI-1 were used as negative and positive controls, respectively. Luminescence signal values divided by OD_600_ values are presented as relative units (RU). The results represent the average values from three independent experiments.

### Western blotting

Samples were separated via SDS-PAGE and transferred to nitrocellulose (pore size: 0.45 μm; Amersham Protran) or PVDF (pore size: 0.45 μm; Amersham Hybond) membranes. These blots were blocked for 1 h in 5% (w/v) skim milk-PBST (0.1% Tween in phosphate-buffered saline [PBS]), incubated for 1 h with appropriate primary antibodies (diluted in 5% skim milk-PBST) at room temperature, washed, and then incubated for 1 h with appropriate secondary antibodies (diluted in 5% skim milk-PBST) at room temperature. Chemiluminescence was detected with EZ-ECL reagents (Cyanagen). The optimal dilution for each antibody was determined as follows: mouse anti-DnaK (Abcam), diluted 1:1000; mouse anti-JNK (BD Pharmingen), diluted 1:1000; and mouse anti-actin (MPBio), diluted 1:10,000. Antibodies directed against T3SS components, including mouse anti-EspB, and mouse anti-Tir, were a generous gift from Prof. B. Brett Finlay (University of British Columbia, Canada) and Prof. Rebekah Devinney (University of Calgary, Canada). Horseradish peroxidase-conjugated (HRP)-goat anti-mouse (Abcam), diluted 1:10,000, was used as the secondary antibody for these analyses. Blots representative of at least three independent experiments are presented in the results section.

### Real-time quantitative polymerase chain reaction (qPCR)

WT EPEC was grown overnight at 37°C in LB broth. The culture was diluted 1:50 into 1:1 (v/v) DMEM: plain LB medium supplemented with either DMSO, CAI-1 (50 μM), or indole (50 or 500 μM) and grown statically in a tissue culture incubator (with 5% CO_2_) for 2 h to the early exponential phase of growth. Bacteria (5 × 10^8^ cells) were collected and RNA was extracted using the NucleoSpin Bacterial RNA isolation kit according to the manufacturer’s guidelines (Macherey-Nagel). RNA was examined for genomic DNA contamination and subjected to additional DNase I treatment when needed, followed by extraction using the TRIzol reagent. A total of 200 ng of RNA from each sample was taken for cDNA synthesis performed using the ProtoScript II First Strand cDNA Synthesis Kit (NEB) using a random primer mix. cDNA was examined for genomic DNA contaminations. Primer sequences used for qPCR are presented in Table 2. Melting curve analyses were used to ensure the specificity of each primer pair. All qPCR analyses were performed using SYBR Green I mix (Roche), sample cDNA, and a LightCycler 480 instrument (Roche) with the following thermocycler settings: 1 cycle at 95°C for 10 min, 40 cycles of 95°C for 15s, cooling to 60°C for 10s, followed by 72°C for 10s. The resultant data were analyzed using the LightCycler 480 software to extract the critical threshold (*CT*) values. The relative expression levels of these target genes following these different treatments were normalized to the *rpoA* housekeeping gene and compared using the relative quantification method. Real-time data are presented as the fold change in expression levels.

### Indole concentration measurement

Indole concentration in the bacterial samples was determined by mixing bacterial supernatants with 20% trichloroacetic acid in a 1:1 (v/v) ratio and incubating them on ice for 15 min. The samples were then centrifuged (at 13,000 × *g* for 10 min) to remove precipitated proteins, collected, and mixed 1:1 (v/v) with Kovac’s reagent (Sigma-Aldrich). The samples were vortexed and left standing for 1 min to allow phase separation. The top layers of the samples were collected, and their optical density at 571 nm was measured. A standard curve of known indole concentration was used to estimate the amount of indole in each sample.

### Translocation assay

Translocation assays were performed as previously described by Baruch *et al*.^41^ with slight modifications. Briefly, EPEC WT and Δ*escN* strains were pre-induced for 3 h under optimal T3SS-inducing conditions or 2 h under semi-optimal T3SS-inducing conditions, statically, in a CO_2_ tissue culture incubator in the presence or the absence of indole (50–1000 μM), CAI-1 (50 μM), or combination of both. EPEC cultures grown in semi-optimal T3SS-inducing conditions were washed prior to infection, to remove the LB medium. HeLa cells (8 × 10^5^ cells/well) were then infected with bacterial cultures at a multiplicity of infection (MOI) of 1:300 for 3 h (when pre-induced under optimal T3SS-inducing conditions) or for 2 h (when pre-induced under semi-optimal T3SS-inducing conditions). The cells were then washed with cold PBS and lysed with RIPA buffer. The lysed samples were collected, centrifuged at 18,000 × g for 5 min to remove unlysed cells, and subjected to western blotting analyses using anti-JNK and anti-actin (loading control) primary antibodies. Uninfected samples and samples from cells infected with the Δ*escN* mutant strain were used as negative controls.

## Supplementary Material

Supplemental Material

## Data Availability

The authors confirm that the data supporting the findings of this study are available within the article, its supplementary material, and are openly available in figshare.com at https://figshare.com/s/00f9b3e28246107780d9.
