# Supplementary material for: Indole intercepts the communication between enteropathogenic E. coli and Vibrio cholerae
Source: Gut Microbes. 2022 Nov 3;14(1):2138677. doi: 10.1080/19490976.2022.2138677 (PMC9635540; doi:10.1080/19490976.2022.2138677)

**Supplementary**

**Bacterial growth:**

EPEC and *V. cholerae* were grown overnight in LB broth, supplemented with the appropriate antibiotics, in a shaking incubator at 37°C or 30°C, respectively. Bacteria were inoculated 1:100 into pre-warmed DMEM medium (WT EPEC) or LB broth (*V. cholerae*) in the presence of either DMSO or 500 µM indole. The absorbance at 600 nm was monitored over time (TECAN Infinity 200Pro plate reader). The results represent average values from three independent biological replicate experiments.

Figure S1A. **Indole does not affect EPEC growth.** Growth curves for WT EPEC grown under optimal T3SS-inducing conditions at 37°C in the absence (○) or presence of 500 μM indole (●). Optical density at 600 nm was measured over time.

Figure S2A. **Indole does not affect *V. cholerae* growth.** **(A)** Pure overnight cultures of EPEC and *V. cholerae* strains were sub-cultured in fresh 1:1 (v/v) DMEM: LB as single or mixed cultures. These cultures were grown in the presence or absence of indole under semi-optimal T3SS-inducing conditions for 6 h. Samples from the cultures were plated on LB plates containing carbenicillin for *V. cholerae* growth. The plates were incubated overnight at 37°C, and bacterial colony-forming units (CFUs) were then counted. To confirm that the lack of T3SS upregulation in the co-culture of EPEC and *V. cholerae* Δ*cqsA* was not due to the low count of *V. cholerae*, we co-cultured the strains with five times (x5) the original inoculation of *V. cholerae* Δ*cqsA* to obtain similar CFUs as the co-culture with WT *V. cholerae*. In parallel, the cultures were separated to bacterial pellets and supernatants (bacterial sup). The secreted proteins were concentrated from collected supernatants and analyzed via 12% SDS-PAGE and western blotting using an anti-EspB antibody. The expression of the effector protein Tir was analyzed by subjecting the bacterial pellets to SDS-PAGE and western blotting using an anti-Tir antibody. Samples were also probed with anti-DnaK to confirm equal loading. **(B)** WT *V. cholerae* was grown in LB broth at 30°C in the absence (○) or presence of 500 μM indole (●). Optical density at 600 nm was measured over time.

Figure S3A. **Indole competes with physiological CAI-1 to influence EPEC T3SS activation.** Wild type (WT) EPEC and *escN* null-mutant (Δ*escN*) EPEC were grown for 6 h under semi-optimal T3SS-inducing conditions in the presence of *V. cholerae* supernatant and various concentrations of indole (50-500 µM). The secreted proteins were concentrated from bacterial culture supernatants (bacterial sup) and analyzed via 12% SDS-PAGE and western blotting using an anti-EspB antibody. The expression of the effector protein Tir, was analyzed by subjecting the bacterial pellets to SDS-PAGE and western blotting using an anti-Tir antibody. Samples were also probed with anti-DnaK to confirm the equal loading of lysates.

Figure S4A. **EPEC, *V. cholerae*, and *B. thetaiotaomicron* growth in single, co-, and tri-culture conditions.** Pure overnight cultures of EPEC and *V. cholerae* strains were sub-cultured in fresh 1:1 (v/v) DMEM: BHI as pure or co-culture. These cultures were grown under semi-optimal T3SS-inducing conditions for 6 h. For tri-culture, *B. thetaiotaomicron* were pre-grown for 8 h in 1:1 (v/v) DMEM: BHI before EPEC and *V. cholerae* were added for additional 6 h growth and then plated on LB plates containing chloramphenicol for EPEC growth (A) or carbenicillin for *V. cholerae* growth (B). The plates were incubated overnight at 37°C, and bacterial colony-forming units (CFUs) were then counted. (C) *B. thetaiotaomicron* were pre-grown for 8 h in 1:1 (v/v) DMEM: BHI before EPEC (co-culture) or EPEC and *V. cholerae* (tri-culture) were added for additional 6 h growth while pure *B. thetaiotaomicron* culture (BT only) was grown for 14 h. The cultures were plated on LB plates containing nalidixic acid, incubated anaerobically for 48 h, at 37°C, and bacterial colony-forming units (CFUs).


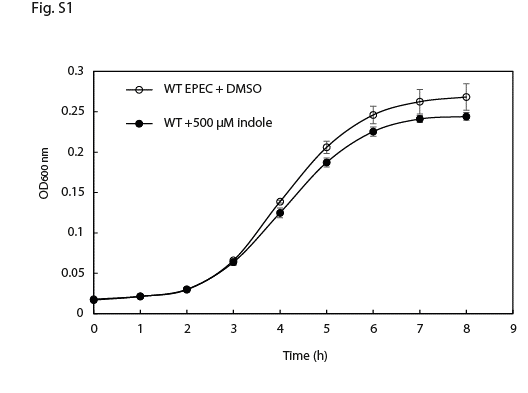


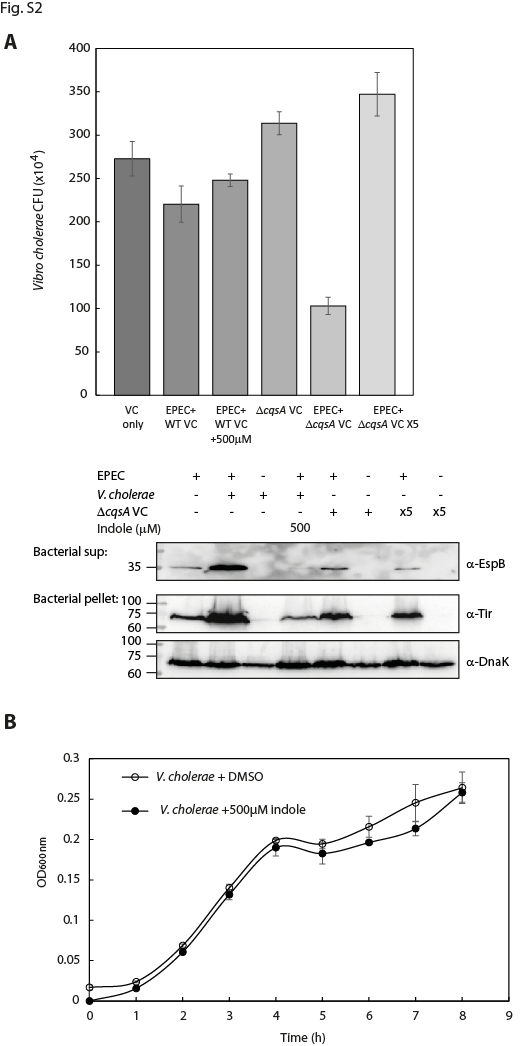


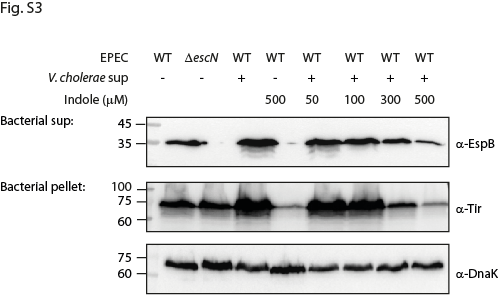


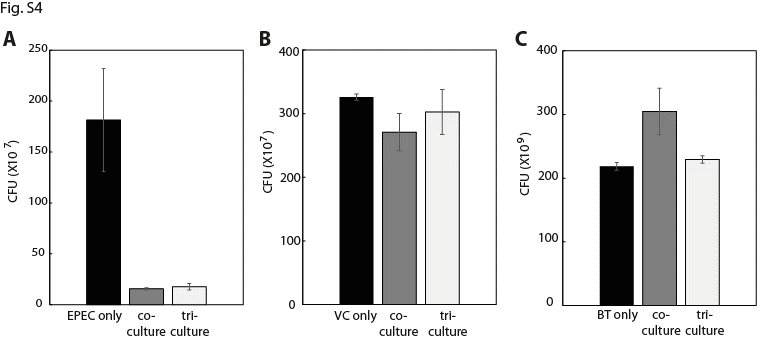

Supplement: Supplemental Material [file KGMI_A_2138677_SM4782.docx]
